# Epidemiology of occupational accidents involving exposure to biological material in Goiânia, Goiás, from 2014 to 2024

**DOI:** 10.47626/1679-4435-2025-1485

**Published:** 2025-12-09

**Authors:** Vítor Gonzaga Chaves, Eduardo William Farinha Carvalho, Gustavo Peres Pelegrine, Stefan Vilges de Oliveira

**Affiliations:** 1 Universidade Federal de Uberlândia, Faculdade de Medicina, Uberlândia, MG, Brasil; 2 Universidade Federal de Uberlândia, Departamento de Saúde Coletiva, Uberlândia, MG, Brasil

**Keywords:** public health surveillance, occupational accidents registry, occupational exposure., vigilância em saúde pública, notificação de acidentes de trabalho, exposição ocupacional.

## Abstract

**Introduction:**

Occupational accidents involving exposure to biological material occur when workers come into contact with blood or other bodily fluids. The literature indicates that most of these events affect health professionals and could be prevented.

**Objectives:**

To describe the epidemiological profile of occupational accidents involving exposure to biological material reported to the Notifiable Diseases Information System in the city of Goiânia, state of Goiás, Brazil.

**Methods:**

This descriptive, cross-sectional study analyzed epidemiological aspects of occupational accidents with exposure to biological material using data from the Notifiable Diseases Information System for Goiânia between 2014 and 2024.

**Results:**

Most reported accidents involved nurses, nursing technicians, or nursing assistants (49.90%), women (76.75%), and individuals aged 20-29 years (37.44%). Among the victims, 580 were not vaccinated against hepatitis B, and 1,004 had unknown or unrecorded vaccination status. Blood was the most frequent biological material involved (75.47%). Loss to clinical follow-up occurred in 17.41% of cases. Two deaths directly related to the accidents were recorded.

**Conclusions:**

There is a need to expand continuing education programs in biosafety for health professionals and to promote large-scale hepatitis B vaccination campaigns. Improving the completeness and quality of reporting is also essential to reduce underreporting of these events.

## Abstract (pt)

**Introdução:**

Acidentes de trabalho com exposição a material biológico envolvem situações nas quais profissionais entram em contato com sangue ou outros fluidos orgânicos. A literatura aponta que a maior parte desses eventos acomete profissionais da saúde e poderia ser evitada.

**Objetivos:**

Descrever o perfil epidemiológico dos acidentes de trabalho com exposição a material biológico notificados ao Sistema de Informação de Agravos de Notificação na cidade de Goiânia, estado de Goiás, Brasil.

**Métodos:**

Estudo descritivo e transversal que analisou aspectos epidemiológicos dos acidentes de trabalho com exposição a material biológico, utilizando dados do Sistema de Informação de Agravos de Notificação referentes a Goiânia, no período de 2014 a 2024.

**Resultados:**

Identificou-se predomínio de acidentes de trabalho com exposição a material biológico envolvendo enfermeiros, técnicos ou auxiliares de enfermagem (49,90%), mulheres (76,75%) e indivíduos entre 20 e 29 anos (37,44%). Entre as vítimas, 580 não estavam vacinadas contra hepatite B, e 1.004 apresentavam status vacinal ignorado ou não registrado. O sangue foi o material orgânico mais frequentemente envolvido (75,47%). Em 17,41% das notificações, houve abandono do acompanhamento clínico. Registraram-se dois óbitos diretamente relacionados aos acidentes.

**Conclusões:**

Há necessidade de ampliar ações de educação continuada em biossegurança para profissionais da saúde e promover campanhas de vacinação em larga escala contra hepatite B. Também se destaca a importância de melhorar a completude e a qualidade das notificações para reduzir a subnotificação dos agravos.

## INTRODUCTION

Occupational accidents involving exposure to biological material refer to situations in which workers from different sectors come into contact with organic materials capable of transmitting pathogens.^1^ Among the most relevant occupational infections are those caused by HIV and the hepatitis B and C viruses.^2,3^ Human blood, for example, can be a source of 20 different diseases.^3^

Health professionals are particularly vulnerable to this type of accident.^4,5^ This increased susceptibility results from performing activities that favor accidental contact with potentially contaminated materials, combined with factors such as long working hours, occupational stress, and, in many cases, insufficient training and inadequate resources.^4-6^

A large proportion of these accidents could be prevented through the adoption of simple biosafety measures.^7^ In addition, even after the event occurs, appropriate clinical follow-up of the victim is essential to prevent infections and late complications.^8^

Although complete elimination of occupational risks is not feasible, occupational medicine seeks to promote workplaces that adopt a culture of prevention, grounded in awareness and good safety practices.^9^ In this context, reporting of accidents is essential, as it enables health authorities to assess and improve mitigation strategies, particularly those related to potentially pathogenic biological agents.^10^

Despite being a legal requirement,^11^ reporting of occupational accidents involving exposure to biological material remains insufficient. Underreporting is influenced by factors such as lack of knowledge among professionals responsible for recording cases, underestimation of severity and infection risk,^12^ and limited understanding of the legal importance of these notifications.^10^

Therefore, this study aimed to identify and describe the epidemiological profile of occupational accidents involving exposure to biological material reported to the Notifiable Diseases Information System (Sistema de Informação de Agravos de Notificação, SINAN) in the city of Goiânia, state of Goiás, Brazil, between 2014 and 2024.

## METHODS

This study is a descriptive, cross-sectional analysis of the epidemiological aspects of occupational accidents involving exposure to biological material in Goiânia.

Data were obtained from SINAN, which is publicly available through the Department of Informatics of the Brazilian Unified Health System. All events reported in Goiânia between 2014 and 2024 were included. Data collection was conducted in February 2025.

The following variables were accessed: number of cases per year, age group, sex, race/skin color, number of pregnant women, educational level, occupation, employment status, outsourced or non-outsourced employment bond, type of exposure, biological material involved, circumstances of the accident, causal agent, use of personal protective equipment (PPE), hepatitis B vaccination status, case outcome, and issuance of a work accident report, which is the official record of an occupational accident or work-related disease. Data were presented in tables as absolute and relative frequencies.

This study relied exclusively on secondary data, without any identification of workers involved in occupational accidents with exposure to biological material, the companies to which they were linked, or the professionals responsible for managing the cases. Therefore, in accordance with National Health Council Resolution no. 510 of April 7, 2016,^13^ submission of the project to a Research Ethics Committee was not required.

## RESULTS

Between 2014 and 2024, 12,490 occupational accidents involving exposure to biological material were reported in Goiânia. The annual number of notifications remained relatively stable over the period, with a mean of 1,135.45 cases per year. Table 1 shows the distribution of cases according to year of notification, sex, and age group of the victims.

**Table 1:** Occupational accidents involving exposure to biological material in Goiânia between 2014 and 2024, according to year of notification, sex, and age group of the victim

Variable/category	Absolute frequency	Relative frequency (%)
Year of notification		
2014	1,174	9.40
2015	1,180	9.45
2016	1,322	10.58
2017	1,231	9.86
2018	1,036	8.30
2019	1,189	9.52
2020	806	6.45
2021	1,090	8.73
2022	1,275	10.21
2023	1,286	10.29
2024	901	7.21
Total	12,490	100.00
Sex		
Male	2,904	23.25
Female	9,586	76.75
Total	12,490	100.00
Age group (years)		
<19	315	2.52
20-29	4,676	37.44
30-39	4,095	32.78
40-49	2,383	19.08
50-59	831	6.66
60-69	171	1.37
70-79	9	0.07
> 80	10	0.08
Total	12,490	100.00

Source: Notifiable Diseases Information System (2025).

The sociodemographic profile of the victims shows a higher incidence of these events among women (Table 1). Among the reported cases, 172 occurred in pregnant women. The 20-29-year age group was the most affected (Table 1). Regarding educational level, most events involved individuals with completed high school (44.51%), followed by those with completed higher education (27.30%) and incomplete higher education (9.63%) (Table 2).

**Table 2:** Occupational accidents involving exposure to biological material in Goiânia between 2014 and 2024, according to victims’ educational level

Educational level	Absolute frequency	Relative frequency (%)
Illiterate	9	0.07
Incomplete 1st to 4th grade of elementary school	110	0.88
Completed 4th grade of elementary school	85	0.68
Incomplete 5th to 8th grade of elementary school	235	1.88
Completed elementary school	254	2.04
Incomplete high school	395	3.16
Completed high school	5,559	44.51
Incomplete higher education	1,203	9.63
Completed higher education	3,403	27.25
Not applicable	120	0.96
Unknown/blank	1,117	8.94
Total	12,490	100.00

Source: Notifiable Diseases Information System (2025).

A higher concentration of cases was observed among health professionals, with accidents involving nurses, nursing technicians, and nursing assistants accounting for approximately 49.90% of all notifications (Table 3).

**Table 3:** Occupational accidents involving exposure to biological material in Goiânia between 2014 and 2024, according to victims’ occupation and employment status

Variable/category	Absolute frequency	Relative frequency (%)
Occupation		
Physicians	1,100	8.81
Students	760	6.08
Nurses	810	6.49
Nursing technicians and assistants	5,422	43.41
Dentists	472	3.78
Pharmacists	211	1.69
Waste collectors	322	2.58
Beauty and aesthetics professionals^*^	123	0.98
Cleaning and maintenance workers†	865	6.93
Physical therapists	123	0.98
Laboratory technicians, assistants, and aides	412	3.30
Other	1,679	13.44
Unknown/blank	191	1.53
Total	12,490	100.00
Employment status		
Registered employee	7,382	59.10
Unregistered employee	360	2.88
Self-employed worker	639	5.12
Statutory public servant	1,226	9.82
Public servant under Consolidation of Labor Laws contract	320	2.56
Retiree	3	0.02
Unemployed	15	0.12
Temporary worker	178	1.43
Cooperative worker	126	1.01
Casual worker	29	0.23
Employer	26	0.21
Other	1,257	10.06
Unknown/blank	929	7.44
Total	12,490	100.00

^*^Including esthetician, manicurist, makeup artist, barber, hairdresser, and podiatrist.

† Including janitor, domestic worker, laundry worker, and housekeeper.

Source: Notifiable Diseases Information System (2025).

Among the victims, 59.10% held registered employment positions (Table 3). Regarding the employment bond of the companies to which the victims were linked, 2,903 workers were outsourced and 7,354 were non-outsourced, while the combined total of the “not applicable” and “unknown/blank” categories in this field of the notification forms corresponded to 2,233 events.

In the analysis of the type of exposure, percutaneous exposure was the most frequent, with 9,962 cases, followed by exposure involving non-intact skin (317 cases), intact skin (1,399 cases), and mucous membranes (1,686 cases). Importantly, the notification forms allow more than one response for this item.

Blood and blood-containing fluids were the organic materials most frequently involved in the incidents (Table 4). The most prevalent circumstances associated with the accidents were intravenous medication administration (9.89%) and the performance of surgical procedures (9.30%) (Table 4).

**Table 4:** Occupational accidents involving exposure to biological material in Goiânia between 2014 and 2024, according to organic material and accident circumstances

Variable/category	Absolute frequency	Relative frequency (%)
Organic material		
Blood	9,426	75.47
Cerebrospinal fluid	121	0.97
Pleural fluid	20	0.16
Ascitic fluid	14	0.11
Amniotic fluid	25	0.20
Blood-containing fluid	584	4.67
Serum/plasma	82	0.66
Other	1,231	9.86
Unknown/blank	987	7.90
Total	12,490	100.00
Accident circumstances		
Intravenous medication administration	1,235	9.89
Intramuscular medication administration	462	3.70
Subcutaneous medication administration	517	4.14
Intradermal medication administration	78	0.62
Blood collection puncture	626	5.01
Unspecified puncture	781	6.25
Improper waste disposal	1,057	8.46
Improper disposal on the floor	1,092	8.74
Laundry activities	105	0.84
Instrument cleaning	472	3.78
Handling of sharps containers	534	4.28
Surgical procedure	1,161	9.30
Dental procedure	693	5.55
Laboratory procedure	364	2.92
Destro	124	0.99
Needle recapping	226	1.81
Other	2,664	21.33
Unknown/blank	299	2.39
Total	12,490	100.00

Source: Notifiable Diseases Information System (2025).

The analysis of the agents involved in the accidents indicates that hollow-bore needles were the main devices associated with the notifications, accounting for 54.91% of the cases.

Among the PPE items, facial protection was the least used, with only 1,324 recorded instances. In contrast, glove use during the event was the most frequently reported, totaling 10,066 notifications. Additionally, 10,906 victims were vaccinated against hepatitis B, 580 were not vaccinated, and in 1,004 forms this information was recorded as unknown or left blank.

Regarding case outcomes, 30 notifications (0.24%) reported discharge with seroconversion and 2,585 (20.70%) reported discharge without seroconversion. In 5,089 notifications (40.74%), this item was marked as unknown or left blank. In 2,606 cases (20.87%), discharge occurred because the source patient tested negative for the biological agent. Loss to clinical follow-up was recorded in 2,175 cases (17.41%). A total of five deaths were reported, two directly related to the accident and three due to other causes.

Concerning the issuance of the work accident report, 7,443 notifications (59.59%) indicated that the document had been issued, while 2,524 (20.21%) reported that it had not. In 2,064 forms (16.53%), this information was recorded as unknown or left blank, and in 459 notifications (3.67%), issuance of the report was marked as not applicable to the case.

## DISCUSSION

The findings of this study showed that health professionals were the main group affected by occupational accidents involving exposure to biological material in Goiânia, a result consistent with previous research.^14,15^ Since health care teams are predominantly composed of women,^15-17^ most victims are expected to be female. Additionally, Vieira et al. reported that male workers tend to underreport accidents involving themselves when compared with women,^10^ which may contribute to the difference observed between sexes.

The high number of notifications involving nursing staff, both technicians and nurses, may be related to the vulnerability of this group, which maintains direct and frequent contact with patients and is responsible for a large portion of routine care procedures.^6,18^ Despite this, evidence indicates that many nursing professionals do not adequately adhere to standard occupational safety measures.^19^

The higher occurrence of accidents among individuals aged 20-59 years, with a progressive decrease in older age groups, as well as the predominance of victims with completed high school education, suggests that professional experience may act as a protective factor. Younger workers with less practical experience may feel less confident when performing high-risk activities,^17^ making them more susceptible to such events.

Routine health care procedures, such as medication administration and surgical interventions, were among the most frequent circumstances associated with the accidents. In this context, the World Health Organization recommends occupational health programs provide continuing education and promote the adoption of safe practices in the workplace.^20^ Training focused on correct handling of sharp instruments is essential in the process,^7,21^ although research still requires methodological refinement in studies evaluating the effectiveness of such interventions.^21^

Proper use of PPE helps reduce exposure to biological agents, protecting both the worker and other staff and patients.^22^ Factors influencing PPE use include availability, the worker’s perception of risk, and the type of employment bond.^23^ In the present study, gloves were the most commonly used PPE, whereas others, such as facial protection and goggles, showed low adherence.

Vaccination against hepatitis B is recommended for susceptible individuals who do not have proof of a complete vaccination schedule or who, even when properly vaccinated, present a non-reactive anti-HBs serology.^24^ Immunization is available free of charge both for health professionals^25^ and for the general population through the Brazilian National Immunization Program.^26^ Since vaccination is not yet universal among Brazilian workers, including those in Goiânia, making it mandatory for higher-risk occupations has been suggested as a relevant strategy.^27^

In the context of labor rights, current legislation establishes that employers are responsible for reporting occupational accidents involving their employees to Social Security; in the absence of this procedure, the report may be filed by the victim or by the attending physician.^28^ It is therefore concerning that a large portion of the notifications analyzed indicated that the work accident report had not been issued. It is important to note that the victim is entitled to an official copy of this document, which carries legal validity to safeguard labor rights.^28^

Finally, a limitation of this study is its reliance on the accuracy of the notifications recorded in the system, which may be affected by human and logistical errors. For example, 105 notifications involved victims reported as younger than 1 year old, an age at which engagement in any form of labor activity is impossible. This highlights the importance of immediate and precise reporting of these events to support timely and effective preventive actions,^29^ and reinforces the need to train health professionals to perform this process properly.

## CONCLUSIONS

The epidemiological profile of occupational accidents involving exposure to biological material in Goiânia highlights the need for a prevention plan tailored to local specificities and to the challenges encountered within the municipal health care network. The findings confirm that health professionals constitute the most vulnerable group due to their continuous exposure to occupational risks. In this context, the implementation of permanent continuing education programs is recommended, with emphasis on biosafety practices, post-exposure management, and the updating of care protocols directed at this population.

The findings concerning the victims’ vaccination status are also concerning, given the substantial number of individuals who were either not vaccinated against hepatitis B or had unknown vaccination status. Thus, the need to intensify hepatitis B vaccination as an essential protective measure is reinforced as well as the importance of ensuring both clinical and occupational follow-up for affected workers.

Finally, the importance of strengthening reporting systems and ensuring transparency of epidemiological data related to these events is underscored, as this supports social oversight and the collaborative development of prevention policies. With the integration of these measures, a significant reduction in accident incidence is expected, along with the consolidation of an institutional culture of occupational safety in health care, in alignment with the organizational principles of the Brazilian Unified Health System.
